# Exosomal nanocarriers of immune fate: molecular insights into cancer immunity, pathogen defense, and emerging immunotherapies

**DOI:** 10.1093/cei/uxag009

**Published:** 2026-02-13

**Authors:** Kamoru A Adedokun, Aminah Bello, Gbadebo M Oyeniyi, Musa K Oladejo, Tajudeen A Adebisi

**Affiliations:** Immunology Department, Roswell Park Comprehensive Cancer Center, Buffalo, NY 14263, USA; School of Biosciences, Faculty of Health and Medical Sciences, Taylor’s University, Subang Jaya, Selangor 47500, Malaysia; Medical Laboratory Science Department, University of Ilorin, Ilorin, Nigeria; Zoology Department, College of Science, King Saud University, P.O. Box 2455, Riyadh 11451, Saudi Arabia; NMC Healthcare, Medical Record and Clinical Documentation Unit, Abu Dhabi 764659, UAE

**Keywords:** exosomes, immune modulation, antigen presentation, pattern recognition receptors (PRRs), extracellular vesicle signaling

## Abstract

Exosomes, the nanoscale extracellular vesicles released by most cell types, are increasingly recognized as potent regulators of immune communication. This review provides a mechanistic and integrative perspective on the immunological functions of exosomes, highlighting their roles in both immune stimulation and suppression across physiological and pathological contexts. We begin by dissecting the molecular architecture of exosomes—focusing on immunologically active components such as ESCRT proteins, tetraspanins, RabGTPases, and lipid mediators—and explore how these elements contribute to exosome biogenesis and immune function. The review further examines exosomal cargo enriched in pattern recognition receptor (PRR) ligands, including damage-associated molecular patterns (DAMPs), pathogen-associated molecular patterns (PAMPs), and microRNAs, and discusses how these molecules activate toll-like receptors and other PRRs to orchestrate innate immune responses through endosomal and cytosolic signaling cascades. Special emphasis is given to MHC-mediated antigen presentation via exosomes, distinguishing classical and non-canonical pathways and their interplay with downstream immune signaling mechanisms. We present a dichotomous view of exosomes as both immunostimulatory and immunosuppressive agents, detailing their roles in T-cell cross-priming, dendritic cell maturation, tumor progression, and metastasis. Moreover, we review pathogen-driven hijacking of exosomal pathways and their implications for immune evasion. Finally, we discuss the therapeutic promise of exosomes in cancer immunotherapy and vaccine design, advocating for their strategic integration into next-generation immunomodulatory approaches.

## Teaser

Exosomes are nanoscale extracellular vesicles that integrate molecular cues to modulate immune responses across cancer and infection contexts. This review dissects mechanistic pathways, including Rab GTPase-TLR signaling crosstalk, non-canonical antigen presentation, and the dichotomy of immunostimulatory versus immunosuppressive exosomes, highlighting their roles in tumor progression, immune tolerance, and pathogen-driven immune evasion. Finally, we explore the emerging therapeutic potential of exosomes as precision immunomodulators and next-generation vaccine platforms.

## Graphical Abstract

**Graphical Abstract:**
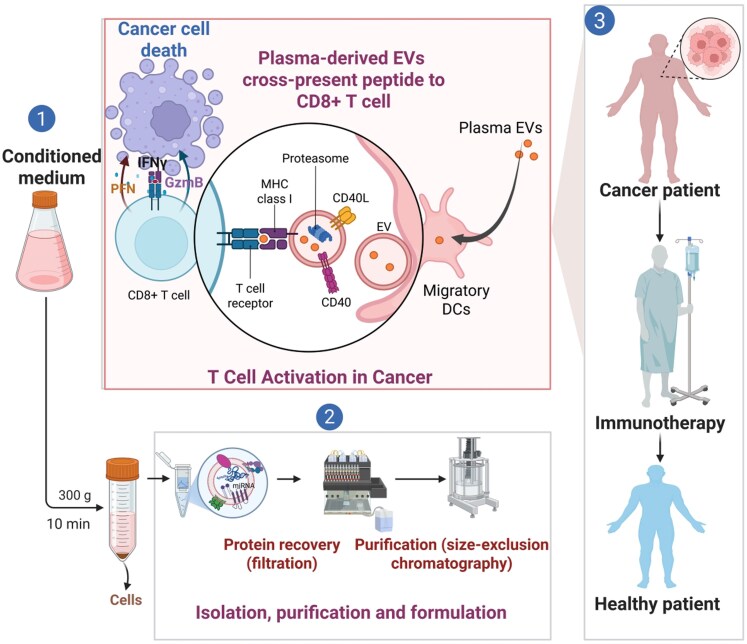
Graphical abstract created in BioRender. Adedokun, K. (2025) https://BioRender.com/rodu9jr

## Introduction

Exosome biogenesis is a tightly controlled process that involves the coordinated action of various components, including proteins, lipids, nucleic acids, and metabolites, resulting in the formation and release of nanoscale extracellular vesicles (EVs). These vesicles remain vital messengers between cells, mediating intercellular communication by moving through body fluids [1]. During the maturation of intraluminal vesicles (ILVs) within multivesicular bodies (MVBs), a wide range of molecules are selectively sorted into these budding vesicles before being packaged into exosomal cargo [2, 3]. The cargo includes proteins (membrane receptors, signaling molecules, enzymes, and tetraspanins such as CD9, CD63, and CD81), lipids (cholesterol, sphingolipids like ceramide, and phospholipids), and nucleic acids (messenger RNA (mRNA), microRNA (miRNA), and DNA). These molecular cargos not only serve as potential therapeutic targets in cancer, autoimmune, inflammatory, and infectious diseases but also play critical roles in cancer immunity by modulating antitumor immune responses, influencing immune cell recruitment, activation, and immune evasion mechanisms within the tumor microenvironment (TME) [1, 4].

The key components of exosomes include the endosomal sorting complexes required for transport (ESCRT) machinery, accessory proteins such as ALG-2-interacting protein X (ALIX), Rab GTPases, tetraspanins, lipids, and diverse protein and nucleic acid cargo [4]. The ESCRT system is essential for the formation of MVBs, which are precursors to exosomes, and for sorting proteins into these vesicles [5]. In cancer, ESCRT components critically regulate the packaging of immunomodulatory molecules, including immune checkpoint proteins such as PD-L1. Exosomal PD-L1 suppresses T-cell activation and proliferation within the TME, thereby facilitating tumor immune evasion and promoting cancer progression [6–8]. Disruption of ESCRT function can alter PD-L1 distribution between the cell surface and exosomes, highlighting potential strategies to restore antitumor immunity.

Tetraspanins, including CD9, CD37, CD63, CD81, and CD151, are transmembrane proteins highly enriched in exosomes and play pivotal roles in exosome biogenesis, cargo sorting, and targeting. In the context of cancer immunity, tetraspanins regulate antigen presentation and co-stimulatory signaling. For example, CD9 facilitates the formation of MHC-II clusters that enhance dendritic cell-mediated T-cell activation, CD37 controls MHC clustering to fine-tune T-cell responses, and CD151 supports co-stimulatory signals required for effective immune surveillance [9, 10]. Through these mechanisms, exosomal tetraspanins help shape antitumor immune responses, influencing T-cell priming, cytotoxicity, and overall immune surveillance in the TME.

Significant knowledge gaps persist regarding the precise mechanisms by which exosomal cargo (e.g. peptide–MHC complexes, lipids, miRNAs, and proteins) modulates innate and adaptive immunity, both in cancer and during pathogen responses. The immunosuppressive versus immunostimulatory roles of exosomes remain putative, particularly in the TME, where exosomal PD-L1 and other immune checkpoint molecules can facilitate immune evasion, and in infections, where pathogen-derived exosomes may either stimulate or subvert host immunity. Several other key unanswered questions exist: the mechanisms governing the selective packaging of immune-related molecules; the roles of tetraspanin-enriched microdomains (TEMs) and ESCRT proteins in loading immunologically relevant cargo; the functional impact of exosomes on diverse immune cell types under normal and pathological conditions; and the contributions of exosomal non-coding RNAs (particularly lncRNAs and circRNAs), as well as exosomal lipids such as ceramide and phosphatidylserine, to immunomodulation. Additionally, the mechanisms of cargo selection, especially regarding immune-related molecules, are highly intricate and remain poorly understood. Addressing these gaps is crucial for fully understanding and harnessing the immunomodulatory potential of exosomes in both cancer and infectious diseases, with implications for immunotherapy and vaccine development.

This study thus begins by describing the molecular architecture of exosomes and their immunomodulatory activities, which include canonical and non-canonical cargo-carrier polymorphisms. It further explores the dual immunological functions of exosomes, shedding light on their contrasting roles in promoting immune activation and facilitating immune suppression. It expands on the intricate dynamics of tumor-derived exosomes in driving immune tolerance, metastasis, and immune evasion, not only in cancer but also in the context of infectious diseases and host–pathogen interactions. By dissecting these mechanisms, the study underscores the complex immunological landscape shaped by exosomes and emphasizes their promise in the development of precision medicine. In closing, it offers a forward-looking perspective on how advancing exosome research may redefine future directions in immunology and immunotherapy.

## Molecular architecture of exosomes: immunological perspectives

### Key proteins and lipids involved in exosome biogenesis as immune-mediating molecules

The precise molecular architecture of exosomes, particularly the specific arrangement and interactions of their key protein and lipid components, dictates their ultimate function in recipient cells, especially immune cells. A deeper understanding of these components is paramount, in the contexts of cancer immunity and host defense, to decipher their roles as regulators of immune signaling, cellular communication, and even immune responses.

**Figure 1:**
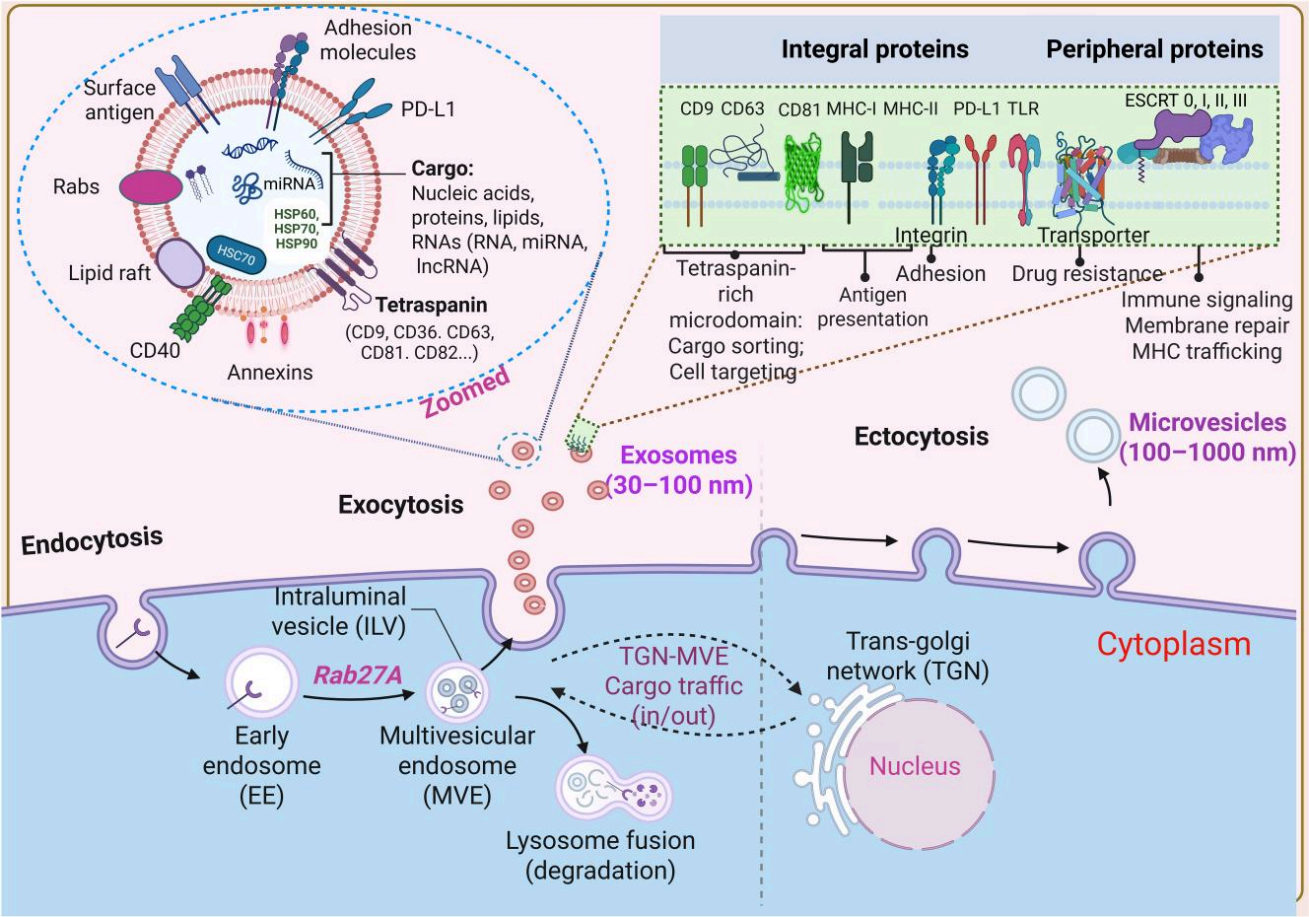
Functional landscape of exosomal membrane proteins: receptors and transporters in targeting, immune modulation, and intercellular communication. Exosomes typically contain cytoskeleton proteins, metabolic enzymes, signal transduction proteins, HSPs, proteins involved in MVB biogenesis (Alix, TSG101, and clathrin), lysosomal-associated membrane proteins (LAMP1 and LAMP2), tetraspanins (CD81, CD9, CD63, and CD82), adhesion molecules (ICAMs and integrins), Rab proteins, and antigen presentation related proteins (CD86, MHC-I and -II). Despite the fact that exosome composition varies, these proteins can be employed as biomarkers to determine whether exosomes are present. Created in BioRender. Adedokun, K. (2025) https://BioRender.com/25b7asc.

Exosomes incorporate a diverse array of membrane-bound and cytosolic proteins, fundamentally shaping their function, as shown in Fig. 1. Key players include proteins involved in membrane transport and fusion, such as Rab GTPases and annexins, alongside those critical to exosome biogenesis, such as the ESCRT complex, ALIX, and TSG101 [11]. Chaperones like HSP70 and HSP90 are also incorporated, as are integrins, and members of the tetraspanin family (such as CD63, CD81, and CD82). Surface expression is richly decorated with numerous antigen and receptor proteins capable of triggering intracellular signaling pathways upon interaction with recipient cells [4]. Further adding to their complexity, exosomes display glycoproteins, glycolipids, cytokines, growth factors, small molecules, and metabolites, all finely tuned to mediate cellular communication.

#### ESCRT and immune mediation

According to a study published by Guo and coworkers, the ESCRT machinery has been implicated in immune cell infiltration into the TME [7]. Phosphorylation of HRS, a key component of the ESCRT complex involved in exosome synthesis, prevents cytolytic CD8 + T cells from infiltrating tumors. HRS interacts with and mediates the preferential loading of PD-L1 to exosomes after ERK-mediated phosphorylation, impeding CD8+ T-cell migration into malignancies.

In another study, the role of ESCRT has also been linked to inflammatory reactions in association with aberrant NF-kB activation. According to Mamińska *et al*. [12], ESCRT components Tsg101, Vps28, UBAP1, and CHMP4B are necessary to regulate constitutive NF-kB signaling. This implies that ESCRTs may play a potential role in preventing spurious activation of NF-kB by constitutively regulating the spatial distribution of cytokine receptors in their ligand-free state [12], suggesting a key role in the mediation of inflammatory processes. Similarly, ESCRT has been largely implicated in autoimmunity. Study shows that mutations in ESCRT components, such as CHMP2B, are linked to neurodegenerative diseases [13] and may influence immune regulation [14]. Defective ESCRT function can impair the degradation of signaling proteins, potentially leading to dysregulated immune activation, contributing not only to autoimmune conditions but also to aberrant immune responses in cancer and during pathogen infections. Beyond the ESCRT machinery, other exosomal components have been linked to a wide range of immunological processes, highlighting their potential as targets or modulators in emerging immunotherapeutic strategies.

#### Tetraspanins and their immune-mediated roles

Tetraspanins are a class of proteins with four transmembrane domains that are involved in important biological functions, including protein trafficking, signaling, membrane fusion, invasion, motility, and cell adhesion [15], many of which are directly implicated in cancer immune responses. Tetraspanins also play a crucial role in exosome biogenesis, cargo sorting, and targeting. They organize membrane microdomains and interact with other proteins, including integrins and signaling molecules. TEMs are strategically organized by widely expressed components such as CD9, CD63, CD81, CD82, and CD151 [16]. Although they are commonly used as exosomal markers, these TEMs affect antigen presentation, target cell uptake, and exosome cargo selection [17]. In the context of antitumor immunity, tetraspanins interact with major histocompatibility complex (MHC) molecules in antigen-presenting cells (APCs) to enhance antigen and T-cell activation. For example, CD9 facilitates MHC-II clustering to improve dendritic cell-mediated T-cell stimulation, CD37 regulates MHC clustering, and CD151 supports co-stimulatory signaling, all of which contribute to effective cytotoxic T lymphocyte responses against tumor cells [17].

The immunogenic potential of EVs, especially their capacity to activate naïve CD4+ T lymphocytes, is believed to be influenced by these interactions presentation [18]. Given these functions, tetraspanin-targeted modification of EV antigen presentation has potential for creating new immunotherapies, such as tumor antigen-loaded exosomes for anticancer responses and cell-free vaccinations. Tetraspanins play a pivotal role in orchestrating antigen presentation. These molecules act as molecular organizers, recruiting diverse proteins to TEMs to form functional complexes critical for immunological synapse (IS) formation. For instance, CD81 facilitates IS assembly, localizing to the central supramolecular activation complex, regulating IS maturation, and interacting with CD3 and ICAM-1 to ensure optimal T-cell activation [19]. Additionally, CD81 is associated with CD4 and CD8 co-receptors, contributing to co-stimulatory signaling that is essential for both effective antitumor cytotoxic responses and pathogen-specific immunity [20]. By modulating TEM organization and exosomal cargo delivery, tetraspanins provide a molecular basis for enhancing immune activation in cancer immunotherapy and infectious disease interventions.

Formation of co-stimulatory signals by CD81 has been repeatedly reported in myriad studies, implicating many unique signaling pathways, particularly in antiviral immunity. Through the nuclear factor-kappa B (NF-κB), NFAT, and AP-1 transduction pathways, tetraspanin CD81 forms a co-stimulatory signal that stimulates the expression of the human immunodeficiency virus type 1 gene in primary CD4+ T cells [21]. Also, in hepatitis C virus, CD81 with CD28 primes naïve T lymphocytes to acquire type 2 effector function [22], suggesting that perturbations of tetraspanin function, such as CD81 deficiency, can impair humoral responses and T-/B-cell activation. Therefore, tetraspanins contribute to the ability of EVs to present antigen and activate naïve CD4+ T cells, as well as play an essential role of EVs with tetraspanins in directing proper degrees of surface clustering required for immune responses.

#### Rab GTPases and the innate immunity responses

Rab GTPases are small GTP-binding proteins that regulate vesicle trafficking. Several Rab GTPases, including Rab27a/b and Rab35, are involved in exosome release [23, 24]. They control the docking and fusion of MVBs with the plasma membrane. Rab27a's role has been specifically highlighted in exosome secretion [24]. Beyond controlling cell vesicle trafficking, which is essential for numerous physiological functions such as mitosis, apoptosis, cell proliferation, and cell nutrition, the role of Rab proteins has been extended to innate immune responses, all of which are central to anticancer immunity. Rab GTPases regulate exosome secretion, which is crucial to the immune system. Rab proteins that are important for exosome formation and secretion include Rab11, Rab35, and Rab27. Rab35 regulates the release of exosomes through its GTPase-activating proteins [25]. Particularly, noteworthy for their role in exosome release are Rab27A and Rab27B. Rab27a and Rab27b are two of the five Rab GTPases that have been found to stimulate exosome secretion in MVE docking [26, 27].

Additionally, Rab31 regulates an exosome route that is not dependent on ESCRT [28]. These results emphasize how crucial Rab GTPases are to the exosome process and how they affect immunological function. While some Rabs affect the endosomal milieu where toll-like receptors (TLRs) dwell, others play a role in the formation of signaling complexes downstream of activated TLRs. Of note, TLRs act as pattern recognition receptors (PRRs) that detect pathogen-associated molecular patterns (PAMPs) or danger-associated molecular patterns (DAMPs), initiating signaling cascades that drive NF-κB, MAPK, and IRF pathways, which are crucial for antitumor immunity, antiviral defense, and immune surveillance.

### PRR signaling ligands (exosomal TLRs, DAMPs, PAMPs, and miRNAs) as immune molecules

Despite the fact that exosomes and TLRs are two separate entities, there is ample evidence suggesting that exosomes can mechanistically regulate innate immunity through interaction with TLRs. Exosomes distribute a variety of biomolecules comprising the ligands for PRRs, including TLRs, which can activate innate immune responses. These ligands include exosomal TLRs themselves, DAMPs, and miRNAs [29].

#### Exosomal TLRs

Exosomes, nanoscale vesicles facilitating intercellular communication, are now established as key modulators of the immune system, particularly through the display of functional TLRs on their surface—a unique mode of intercellular communication. This enables direct activation of immune cells, circumventing traditional antigen presentation pathways and offering a rapid response mechanism.

**Figure 2:**
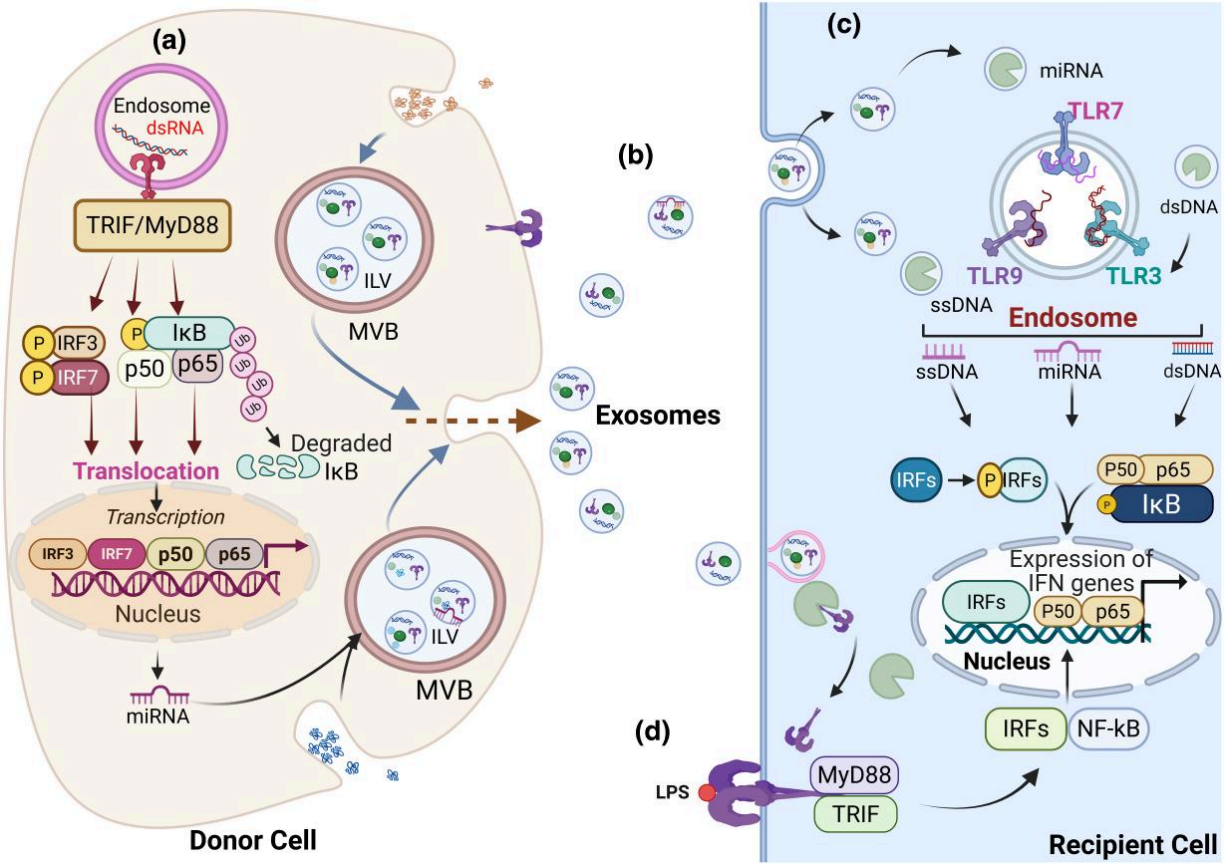
Exosome-mediated trafficking of pattern recognition ligands and their engagement of endosomal and surface TLRs: a hub for IRF and NF-κB signaling (a) Cytosolic/nuclear dsDNA accumulation (e.g. tumor, stress, and damage, infection)—trafficking to endosome (via autophagy-like or vesicular pathways) [not shown]—endosomal TLR9 activation (binds CpG-rich dsDNA)—MyD88—IRF3,7/NF-kB activations: (i) IRF3,7→ Type I IFNs (IFN-α and IFN-β): miRNA induction (e.g. IFN-inducible miRs like miR-155 and miR-146a) → packaging into exosomes → exported to recipient cells (ii) NF-kB activation → pro-inflammatory cytokines (e.g. IL-6 and TNF-α). (b) Donor cell (exosome released) → [exosome + miRNA/DNAs] encapsulated → recipient cell (via endocytosis) → TLR7/8 or RISC pathways. (C) Exosomal ssDNA: Donor tumor/immune cell → exosome (encapsulated ssDNA) → recipient dendritic cell → [endocytosis → endolysosome] → TLR9 activation → IRF7 + NF-κB signaling → IFN-α, TNF-α production; Exosomal miRNAs (e.g. miR-21, miR-29a): Donor tumor cell → exosome (miR-21/29a) → recipient immune cell (mouse DCs or human MoDCs) → [endocytosis → endosome] → TLR7 (mouse)/TLR8 (human) → MyD88-Dependent NF-κB Pathway → IL-6, TNF-α, type I IFNs; Exosomal dsDNA (e.g. mtDNA or viral DNA): Tumor/virus-infected cell → [exosome (dsDNA)] → recipient pDCs/macrophages →; (i) endosomal route: dsDNA → TLR9 → IRF7 + NF-κB → IFN-α/β, or (ii) → cytosolic route: dsDNA escape → cGAS-STING Pathway → Type I IFNs. (d) Through MyD88 and TRIF signaling, TLRs, such as TLR4 within exosomes, can be transported from donor cells to recipient cells, resulting in TLR-4 functioning cells that are responsive to LPS. Created in BioRender. Adedokun, K. (2025) https://BioRender.com/kplhpvj.

For instance, a study shows that TLRs are transferred between dendritic cells by EVs [1]. The investigators explain that bone marrow-derived dendritic cells (BMDCs) adopt EVs to transfer functional TLR4 to TLR4-knockout (TLR4KO) BMDCs. By internalizing these EVs, TLR4KO BMDCs effectively reverse their non-responsive phenotype by becoming responsive to lipopolysaccharide (LPS) again, triggering the NF-κB signaling pathway and generating pro-inflammatory cytokines such as tumor necrosis factor (TNF)-α and IL-6, as further described in Fig. 2.

Collectively, Zhang *et al*. [1], reveal a non-canonical mechanism of innate immune amplification, whereby EVs horizontally transfer functional TLR4 to recipient dendritic cells, reprogramming their responsiveness to LPS and reinstating NF-κB–dependent inflammatory cytokine production.

Typically, in canonical TL signaling, TLR signaling is initiated when a TLR on the cell surface or in an endosome binds to a specific PAMP or DAMP ligand. This ligand binding triggers a cascade of intracellular signaling events, including recruitment of adaptor proteins (e.g. MyD88 and TRIF), activation of kinases, and nuclear translocation of transcription factors (e.g. NF-κB and IRF3) to induce the expression of target genes involved in immunity and inflammation. In the canonical scenario, exosomes might be involved in delivering PAMPs or DAMPs to TLRs on recipient cells.

So, the exosomes are the cargo carriers delivering the active molecule (the ligand) to the target cell, where a cellular response, with TLRs located on this responding cell, is observed. In this study, however, the exosome transfers functional TLR4 to TLR4KO cells, providing them with the receptor they were missing. The exosome, in a way, reconstitutes the TLR4 signaling pathway in the recipient cell. The ligand (LPS) is then delivered ‘independently’ after exosome transfer.

Critical areas for future investigation include elucidating the precise mechanisms of TLR sorting into exosomes, the role of lipid raft association in TLR signaling on exosomes, and the potential for targeted manipulation of exosomal TLR expression to modulate immune responses in disease. Characterization of these mechanistic details will be crucial for therapeutic exploitation of exosomal TLRs in cancer immunotherapy, vaccine development, and treatment of inflammatory diseases.

#### Danger-associated molecular patterns/pathogen-associated molecular patterns

Exosomes can also carry DAMPs, such as HMGB1, S100 proteins, and heat shock proteins (HSPs), which are released from damaged or stressed cells. DAMPs can activate PRRs, such as TLRs and the receptor for advanced glycation end products, leading to inflammation and immune activation. This is particularly feasible in antitumor immunity. Similarly, PAMPs are other elements of exosomal cargo that have been found crucial for the induction of an immune response, especially in infectious diseases. For instance, as demonstrated by Liu *et al*. [30], exosomes derived from *Mycobacterium tuberculosis* (M.tb)-infected mesenchymal stem cells (MSCs)-derived exosomes (Exo-MSCs-M.tb) are enriched with 19-kDa lipoprotein and lipoarabinomannan (LAM). Mechanistically, when these Exo-MSCs-M.tb are taken up by macrophages, the 19-kDa lipoprotein and LAM act as ligands that bind to TLR2/4 on the macrophage surface. This binding activates the TLR2/4 signaling pathway, leading to the recruitment of MyD88, subsequent activation of NF-κB and MAPK pathways, and ultimately, the pro-inflammatory response of macrophages, marked by increased production of TNF-α, RANTES, and inducible nitric oxide synthase (iNOS).

#### microRNAs

Exosomes are rich in miRNAs, small non-coding RNAs that regulate gene expression. Exosomal miRNAs can be delivered to recipient cells, where they can modulate gene expression and influence immune responses. For example, exosomes can carry miRNAs that target mRNAs encoding inflammatory cytokines, suppressing inflammation. About two decades ago, research extensively documented the presence and function of miRNAs within exosomes and their regulatory roles as a unique way of genetic exchange across cells [31].

Similarly, recent investigations have highlighted the role of microRNAs in cancer progression. Ascites-derived exosomes (ADEs) from ovarian cancer patients have been shown to promote metastasis by inducing epithelial–mesenchymal transition (EMT) in ovarian cancer cells through the transfer of microRNA-6780b-5p (miR-6780b-5p) [32]. This effect occurs after ADEs are taken up by recipient cells, where the miR-6780b-5p downregulates the expression of E-cadherin, and upregulates N-cadherin and Vimentin, promoting EMT, which consequently enhances ovarian cancer cell migration, invasion, and proliferation, and thus, facilitates metastasis.

### Rab GTPases meet TLR signaling: orchestrating innate immunity through intracellular trafficking

Rab GTPases play intricate roles in regulating the TLR signaling pathway, a cornerstone of innate immunity. TLRs, essential for recognizing PAMPs, are strategically localized: TLR2 and TLR4 reside on the cell surface, while TLR3, TLR7/8, and TLR9 are predominantly found within endosomes. This compartmentalization is critical for detecting diverse PAMPs.

Upon ligand binding, TLRs initiate signaling cascades via their Toll/interleukin-1 receptor domain, recruiting downstream adaptors. These adaptors, in turn, activate Interferon Regulatory Factor 3/7 and NF-κB, leading to the production of type I interferons (IFN-I) and TNF, key cytokines in antiviral and inflammatory responses [33] (Fig. 2). Rab GTPases are crucial for the intracellular trafficking of TLRs between the cell surface and endosomes, thereby controlling their accessibility to ligands and modulating the strength and duration of TLR signaling. Some Rabs participate in the assembly of signaling complexes downstream of activated TLRs, while others influence the endosomal environment where TLRs reside, thereby affecting downstream signaling. Thus, dysregulation of Rab function can lead to aberrant TLR signaling, contributing to chronic inflammation or impaired immune responses.

### MHC-mediated antigen presentation and co-stimulation by exosomes

#### Non-canonical MHC-mediated antigen presentation in exosomes

Exosomes offer a distinct mode of antigen presentation compared to the conventional *de novo* pathway. Rather than synthesizing MHC molecules and loading them with processed peptides within cellular compartments, exosomes primarily display pre-formed MHC-peptide complexes acquired from their originating cell. The current understanding posits that these mature complexes, already processed via cellular antigen presentation machinery, are selectively sorted into MVBs during exosome biogenesis [34]. The ESCRT machinery facilitates this selective packaging into ILVs [35], while lipid rafts and tetraspanins contribute to MHC molecule stability and clustering on the exosome surface [36, 37]. This non-canonical presentation means that exosomes act as carriers of established antigen-MHC interactions, reflecting the immune status of the cell from which they originate rather than actively processing new antigens.

Exosomes have been discovered to possess diverse types of proteins on their surface, which include: T- and B-cell receptors (BCRs), lectins, cytokines, cytokine receptors, and integrins, which may provide specificity for recognition and binding to target cells [38]. These exosomes, after binding, are internalized and their contents released either through direct fusion with the plasma membrane or as intact vesicles via the endosomal pathway. This distinction is especially significant in APCs, which internalize exosomal antigens and process them within endosomal compartments, where they are loaded onto MHC molecules and subsequently presented to effector lymphocytes, thereby initiating an immune response [39]. Molecular profiling has shown that exosomes typically contain intraluminal HSPs, tetraspanins, MVB biogenesis proteins, as well as lipid-related proteins and phospholipases [40]. However, those generated from immune cells were rich in immune-related proteins, such as antigen-presenting molecules (MHC class I, MHC class II, and CD1), cell adhesion molecules (CD11b and CD54/ICAM-1), and co-stimulatory proteins (CD86) [39].

An earlier study by Clayton *et al*. [41]. utilizing immuno-magnetic isolation and flow cytometry characterized exosomes derived from B lymphocytes, revealing high surface expression of MHC class I and II molecules. Furthermore, these exosomes displayed immunologically relevant co-stimulatory markers B7-1 (CD80) and B7-2 (CD86), along with the adhesion molecule ICAM-1 (CD54). Confirming their B-cell origin, the exosomes also expressed CD20 and the complement regulatory protein CD59. Notably, the lysosomal marker CD63 exhibited variable expression, while the transferrin receptor (CD71) was undetectable.

Likewise, exosomes secreted by monocyte-derived dendritic cells, cultured for 7 days in (granulocyte-macrophage colony-stimulating factor) GM-CSF and IL-4 to maintain an immature phenotype, exhibited a similar molecular profile with abundant MHC molecules [42]. CD63 expression was consistently strong in these exosomes, and the presence of CD1a, an MHC Class I-like molecule, suggested a potential role in lipid antigen presentation. CD59 was also expressed, indicating a possible function in complement regulation. However, markers such as CD71, CD40, CD14, CD20, and CD83 were not detected [41]. This variable expression of markers such as CD63, coupled with the absence of CD71 in different exosomes of immune cell origin, suggests potential heterogeneity in their cargo contents and implies that not all exosomes carry the same protein composition, emphasizing the need for careful characterization in functional studies.

Besides, the ability of exosomes to present pathogen-derived peptides has been demonstrated for both bacterial and viral antigens [43]. Upon activation with LPS, a component of Gram-negative bacteria, dendritic cells interacting with non-cognate activated T cells undergo morphological changes, triggering the release of exosomes. These exosomes are enriched with MHC class II peptide complexes, ICAM-1, and notably, elevated levels of miR-155a, a critical regulator of T-cell responses known to activate antigen-specific CD8+ T cells [44]. This suggests a mechanism by which activated DCs can amplify T cell-mediated immunity via exosome-mediated transfer of key signaling molecules and regulatory miRNAs.

In another study, dendritic cells exposed to *Escherichia coli* produced enormous quantities of EVs containing MHC class II molecules and bacterial antigens derived from the phagocytosed microbes [45]. This process likely involves the inward budding of ILVs from the phagosomal membrane, enabling the incorporation of bacterial components. The findings highlight the potential of these exosomes to effectively present antigens and stimulate adaptive immune responses when loaded with pathogen-derived peptides presented on MHC molecules [46]. Exosomes derived from B cells have been discovered to contain MHC-I and MHC-II molecules, CD20, CD45, and BCR complexes, which include surface immunoglobulin, CD19, and tetraspanins, along with chaperone proteins like HSP-70 and -90, integrins, as well as various other associated proteins [47].

**Figure 3:**
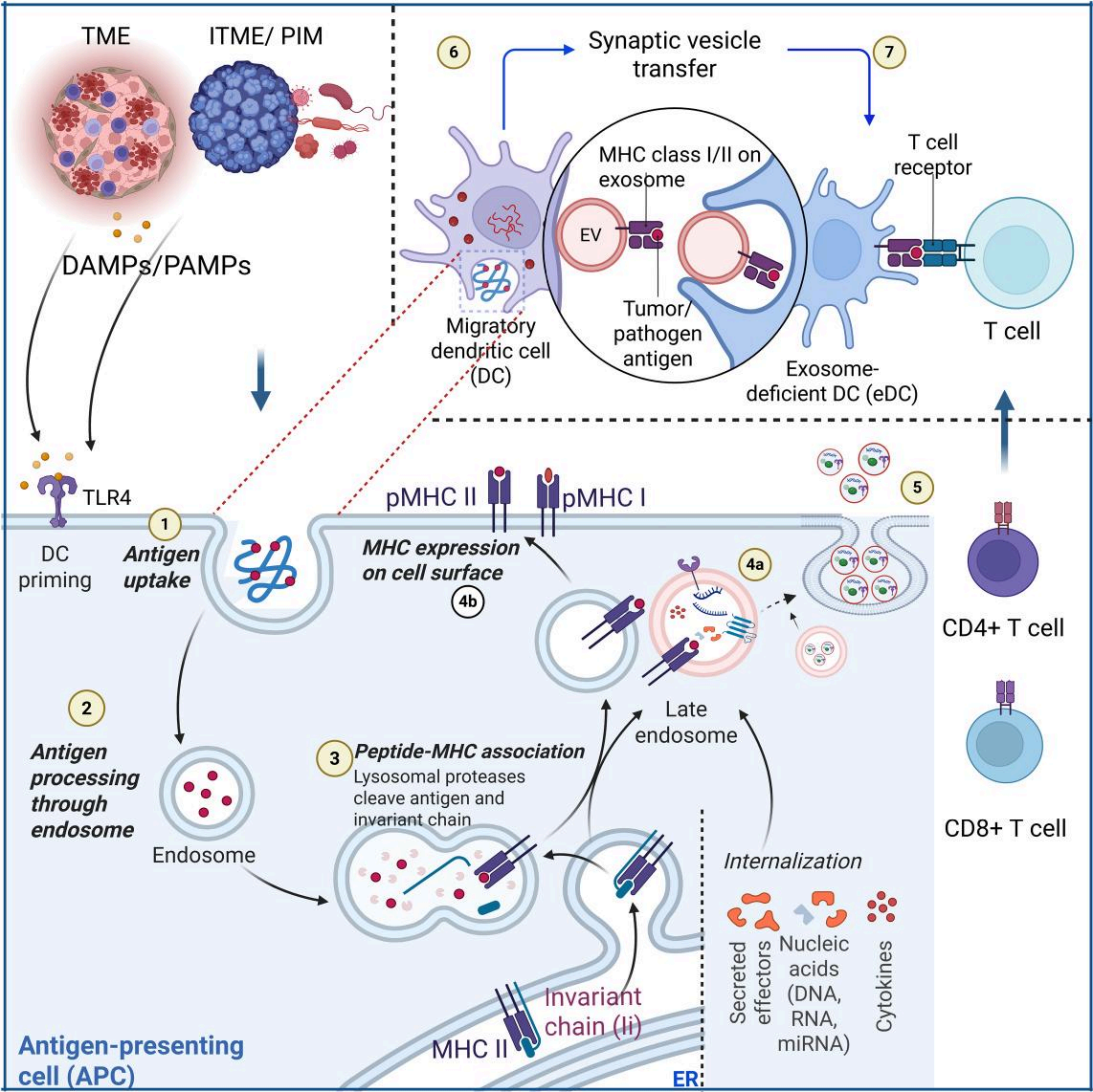
Exosome-mediated coordination of MHC-I and MHC-II antigen presentation and cross-priming: from donor DCs to exosome-deficient DCs. TME: tumor microenvironment; ITME: infected tissue microenvironment; PIM: pathogen-infected microenvironment. EV: extracellular vesicle; pMHC: peptide–MHC complex; DC: dendritic cell; DAMPs: danger-associated molecular patterns; PAMPs: pathogen-associated molecular patterns. 1. Antigen uptake by antigen-presenting cells (APCs): Upon prior stimulation by damage-associated molecular patterns (DAMPs) or pathogen-associated molecular patterns (PAMPs) (e.g. TLR ligands or tumor debris), exosome-donor dendritic cells become primed and activated. These DCs then engulf antigens via phagocytosis, receptor-mediated endocytosis, or macropinocytosis, often sourcing antigens from tumor cells, infected cells, or apoptotic bodies. 2. Endosomal routing and cytosolic transfer: Internalized antigens are routed into early endosomes, where they may follow different processing routes. For MHC-I cross-presentation, antigens are transferred into the cytosol, degraded by the proteasome, and re-routed to the ER or recycling endosomes. For MHC-II presentation, antigens remain within acidic endo-lysosomal compartments for enzymatic processing. 3. Peptide–MHC association: In the MHC-I pathway, short peptides generated by the proteasome are transported via TAP into the ER and loaded onto MHC-I molecules. In the MHC-II pathway, peptides generated in MIIC compartments bind to MHC-II after degradation of the invariant chain (Ii). 4a. Surface expression of MHC-peptide complexes: loaded MHC-I and MHC-II molecules are transported to the DC plasma membrane for antigen display to T cells. 4b. Cargo buildup of MHCs and internalization into exosome precursors: In parallel with surface expression, both MHC-I and MHC-II molecules, along with other cargoes such as miRNAs, cytokines (e.g. IL-6 and IFN-β), immunoregulatory molecules (e.g. CD86 and PD-L1), and nucleic acids (e.g. dsDNA), are selectively incorporated into ILVs within multivesicular endosomes (MVEs) via ESCRT-dependent or ceramide-mediated pathways. 5. Release of exosomes by migratory DCs: Upon maturation, migratory donor DCs traffic to lymphoid tissues, where MVEs fuse with the plasma membrane, releasing exosomes enriched with immunogenic and regulatory molecules, including MHC–peptide complexes. 6. Synaptic transfer of exosomes to exosome-deficient DCs (eDCs): In the lymph node microenvironment, donor DC-derived exosomes are transferred across immunological synapses to bystander eDCs. This horizontal transfer enables eDCs to acquire functional MHC-I and MHC-II–peptide complexes, bypassing the need for antigen uptake. 7. T cell cross-priming involving CD8^+^ and CD4^+^ T cells: CD8^+^ T cells are activated through MHC-I-peptide presentation, acquiring cytotoxic function against infected or malignant targets. CD4^+^ T cells are primed via MHC-II–peptide complexes, providing essential help for cytotoxic T-cell memory formation and B-cell activation. This inter-DC exosome transfer enables efficient cross-priming of both arms of the adaptive immune system, even when antigen presentation is compartmentalized or limited to a subset of DCs. Created in BioRender. Adedokun, K. (2025) https://BioRender.com/kplhpvj.

Exosomes carrying MHC-peptide complexes or antigens can function as indirect platforms for antigen presentation after being taken up by other cells. Research has shown that both migrating and resident DCs can exchange antigens via exosomes, enabling DCs that have not directly encountered the antigen to still contribute to T-cell priming, a process known as cross-priming [48]. Once EVs are internalized, their antigens may enter the endosomal pathway, where they are degraded and presented on MHC class II molecules, as shown in Fig. 3. Alternatively, the antigens can be released into the cytoplasm, processed, and presented on MHC class I molecules. This mode of antigen transfer enhances the overall capacity for antigen presentation and improves the efficiency of T-cell activation [49]. Although most of the literature focuses on pathogen-derived antigen presentation, similar mechanisms may operate in the context of tumors. Tumor-derived exosomes can carry tumor-associated antigens (TAAs) in complex with MHC molecules, tetraspanins, and co-stimulatory proteins, potentially enabling cross-priming of naïve T cells by dendritic cells that have not directly encountered the tumor. By analogy to pathogen-derived exosomes, this suggests a mechanism whereby tumor EVs may enhance antitumor immunity, stimulate CD8 + cytotoxic T-cell responses, and modulate the TME, providing a functional parallel between microbial and tumor antigen presentation.

#### Exosomes vs. trogocytosis: non-canonical but distinct concepts

Exosomes present a non-canonical mode of antigen presentation that contrasts with traditional *de novo* processing and, importantly, with trogocytosis. In the context of MHC-mediated antigen presentation, trogocytosis describes a process where one immune cell, particularly dendritic cells, acquires MHC-peptide complexes directly from the surface of another cell through cell–cell contact, enabling the acquiring cell to display those antigens as “self” [50]. Beyond DCs, this MHC transfer via trogocytosis occurs between various immune and non-immune cells, sometimes effectively conferring antigen-presenting activity even on non-professional APCs [51]. While both exosomes and trogocytosis facilitate intercellular transfer of MHC-peptide complexes, their underlying mechanisms and functional consequences differ. Exosomes, formed via the ESCRT-mediated pathway, selectively package pre-formed MHC-peptide complexes from their cell of origin into ILVs within MVBs [34, 45]. In contrast, trogocytosis involves the direct acquisition of membrane fragments, including MHC molecules, from one cell by another through cell–cell contact, without the intermediary step of exosome biogenesis. While some instances of exosome transfer and trogocytosis may be mediated via a shared mechanism [50], trogocytosis often involves a broader, less selective transfer of membrane components, whereas exosomes can be enriched for specific cargo. The resulting functional differences suggest that while trogocytosis enables rapid, albeit less targeted, antigen display, exosomes provide a more controlled and targeted mechanism for shaping immune responses.

## Mechanistic insights into immune-stimulatory and immunosuppressive exosomes: an immunoregulatory dichotomy

### Overview of the immune function of exosomes

Recent studies have shown that exosomes generated from host cells, tumor cells, bacteria, and parasites facilitate communication between invading pathogens and innate immune cells, thereby playing a crucial role in the spread of pathogens and donor cell-derived molecules, as well as in regulating the host's innate immune responses [18, 29, 30, 39, 43, 54]. Two distinct modes of exosomal immune regulation are described below: immunostimulatory and immunosuppressive functions.

#### Immunostimulatory role of exosomes

Exosomes possess immune-activating properties. Exosomes activate the immune system via antigen presentation as they can function as antigen-presenting vesicles that activate T- and B-cells, leading to the induction of either cellular or humoral adaptive immune responses [52]. Exosomes, originally identified in B lymphocytes utilizing MHC class I and II proteins for antigen presentation, have since been shown to mediate diverse immunostimulatory functions across various immune cell types [53]. To avoid redundancy, the concept of antigen presentation by exosomes was previously detailed (see section MHC-mediated antigen presentation and co-stimulation by exosomes).

Additionally, exosomes sometimes bear components such as DNA strands, viral RNA and proteins, which can stimulate the release of cytokines, thereby modulating innate immune response [54]. Beyond protein-mediated antigen presentation, exosomes encapsulate DNA that reflects the genetic landscape of the originating cell [55]. Notably, exosomal DNA levels distinguish healthy individuals from cancer patients, suggesting diagnostic potential [56]. Furthermore, the immunostimulatory capacity of exosomal DNA extends to innate immune responses, as exemplified by topotecan treatment in breast cancer, which triggers DNA double-strand breaks, enhances exosomal DNA production, and subsequently activates dendritic cells through cGAS-STING signaling [57]. Collectively, these findings implicate exosomes as potent modulators of both adaptive and innate immunity. The precise mechanisms governing DNA packaging into exosomes remain an exciting avenue for future research, promising further insights into targeted immune activation strategies.

#### Immunosuppressive role of exosomes

Exosomes also wield potent immunosuppressive capabilities, making them attractive therapeutic tools for autoimmune diseases and transplantation tolerance. A key mechanism involves CD4^+^CD25^+^ regulatory T cell (Treg)-derived exosomes, which selectively package miRNAs and iNOS. Upon delivery to target T cells, this cargo disrupts cell cycle progression, promotes apoptosis, inhibits proliferation, and even induces the differentiation of immature T cells into Tregs, thereby amplifying the immunosuppressive milieu. This is further augmented by high CD73 expression on Treg exosomes, enhancing adenosine generation and immune quiescence [58].

Moreover, exosomes can directly suppress T-cell function through the transfer of inhibitory membrane proteins such as PD-L1, which engages PD-1 receptors on T cells to impair activation and promote tumor immune escape [59, 60] Emerging technologies like the EXID system facilitate the detection of exosomal PD-L1, serving as a valuable marker of immune resistance [61]. Illustrating this further, syncytiotrophoblast-derived exosomes express a potent combination of immunosuppressive molecules, including NKG2D ligands (MICA/B and ULBP1), death ligands (FASL and TRAIL), and immune checkpoint ligands (PD-L1 and PD-L2), to establish and maintain maternal–fetal immune tolerance [18, 39]. This regulatory action extends to graft rejection, as evidenced by WJMSC-derived exosomes suppressing T cells via PD-L1 [54, 58, 59] and underscores that blocking PD-L1 secretion from exosomes can significantly enhance antitumor immunity [62].

This dichotomous understanding underscores the plasticity of exosomes as both immune suppressors and stimulators, with implications for cancer immunotherapy, maternal–fetal health, and transplant medicine.

Of note, beyond simple activation or suppression, exosomes actively shape the inflammatory landscape. In systemic lupus erythematosus (SLE), for example, disease-specific exosomes potently stimulate the production of pro-inflammatory cytokines like IFN-α, IL-1β, and IL-6 [63]. Notably, in the absence of exosomes, cytokine production in SLE serum diminishes significantly, suggesting a direct link between exosome levels and disease activity. In broader contexts, exosome-associated proteins and miRNAs exhibit dysregulated expression patterns in autoimmune diseases, contributing to both the promotion and suppression of inflammation [64]. This suggests a complex, context-dependent role for exosomes in fine-tuning the inflammatory response.

### Exosome involvement in T-cell priming, B-cell activation, and dendritic cell maturation

The process by which a naïve T cell is activated and undergoes clonal expansion upon first encountering an antigen presented on the surface of an APC is commonly referred to as T-cell priming [65].

Dendritic cell exosomes (DEX) carry antigen-presenting molecules such as MHC class I and II, CD80, CD86, and HSPs Hsp70 and Hsp90, which can stimulate T cells through both direct and indirect mechanisms. In the direct pathway, T cells are activated by MHC molecules and co-stimulatory signals displayed on the surface of DEX. However, this method is generally ineffective in activating naïve T cells due to insufficient T-cell receptor (TCR) engagement and co-stimulatory signaling. In contrast, indirect activation is more efficient in stimulating naïve T cells, as bystander dendritic cells can uptake antigens from DEX and then effectively prime antigen-specific T cells. Additionally, DEXs can enhance T-cell responses against tumors by transferring MHC-peptide complexes to the surface of tumor cells [66].

Dendritic cell-derived exosomes can also promote the maturation of bystander dendritic cells by binding to them and cross-presenting TLR ligands. This interaction increases the expression of transmembrane TNF and boosts the secretion of key pro-inflammatory and immunomodulatory cytokines, thereby effectively triggering a strong innate immune response and promoting Type 1 T-helper cell (Th1) polarization [67].

Exosomal MHC-II molecules are mainly found in secondary lymphoid organs and are expressed only by specialized antigen-presenting cells, including B cells. Exosomes are also found in tertiary lymphoid structures due to the presence of B cells. B cells are activated through calcium channels as a result of CD20 present on their membrane, which in turn results in stimulated calcium channels leading to release of numerous exosomes [68].

B cell-derived exosomes present peptide–MHC class II (pMHC-II) complexes on their surface. The concept that these exosomes can carry pMHC-II was proposed over two decades ago and has since been validated. Most primary B-cells release exosomes that display pMHC-II. When antigen-loaded B-cells engage with specific T cells, they become activated, leading to increased exosome release and protection of pMHC-II from intracellular degradation. These pMHC-II complexes interact with TCRs on naïve CD4+ T cells, triggering their activation and initiating an immune response. In contrast, exosomes derived from mature dendritic cells are relatively inefficient at activating naïve T cells [68]. Exosomes released by B lymphocytes are crucial for activating and sustaining primed CD4+ T cells. Research indicates that activated T-cells promote the release of exosomes from B cells, which then enhance T-cell proliferation and differentiation [69].

*In vitro* studies have shown that exosomes released by B cells can specifically bind to follicular dendritic cells (FDCs). The survival and function of B cells within germinal centers rely on their adhesion to FDCs via the VCAM-1 pathway. Notably, FDCs do not naturally express MHC class II molecules; instead, they acquire peptide-loaded MHC-II from B cells. The interaction between B cell-derived exosomes and FDCs may occur through VCAM-1, potentially promoting T helper cell activation. In tumor environments, FDCs secrete the chemokine CXCL13, which effectively attracts lymphocytes, including B cells. Based on this, it is suggested that in tumors, FDCs may recruit B cells that interact with exosomes, facilitating antigen presentation. Therefore, FDCs may serve as a key physiological target for B cell-derived exosomes in immune responses [68].

Mast cell-derived exosomes carry external antigens in complex with HSPs HSP-60 and HSC-70, enabling them to perform various immune functions depending on the context. These exosomes can activate both B and T lymphocytes *in vivo* and *in vitro*. The external antigens within the exosomes are delivered to T cells through a process called cross-presentation, mediated by dendritic cells. DCs capture the HSP-60 or HSC-70-antigen complexes via the endocytic receptor CD91. After internalizing and processing the antigens, DCs present them on their surface through the MHC-II pathway. Additionally, the presence of HSP-60 and HSC-70 in mast cell exosomes promotes the upregulation of co-stimulatory molecules such as CD80, CD86, and CD40 upon interaction with DCs. This interaction enhances DC maturation and improves their capacity for antigen presentation [70].

## The immunological impact of exosomes: from immunosuppression to metastasis

The content of exosomes, including proteins, lipids, nucleic acids, and cytokines, allows them to play roles in immune system modulation and other complex biological processes. While tumor-derived exosomes carry immunosuppressive factors for tumor progression and metastasis, Treg exosomes mainly use them to maintain immune homeostasis.

### Roles of tumor exosomes in immunosuppression and metastasis

Tumor exosomes are able to suppress innate immunity by reducing the surface expression of the receptor NKG2D, thereby diminishing NK cells’ capacity to recognize NKG2D ligands on tumor cells and lyse them [64]. Furthermore, in NK cells and CD8+ T cells, tumor exosomes impair IFN-γ production and cytokine release [71]. miR-92b and miR-23ab carried by tumor exosomes have been linked to reduced NK Cell cytotoxicity [72].

The adaptive immune system is also a target of tumor exosome-mediated immunosuppression. These exosomes act as vehicles for immunosuppressive cytokines TGF-β and IL-10, which inhibit the expression of key co-stimulatory molecules CD80 and CD86 on dendritic cells. This results in reduced DC activation and, consequently, reduced T-cell activation. Simultaneously, these cytokines promote the differentiation and activation of Treg cells, tilting the balance toward an immunosuppressive state [73, 74]. Other than cytokines, tumor exosomes can deliver messenger RNAs, proteins and microRNAs that alter gene expression in immune cells to immunosuppressive phenotypes, while activating myeloid-derived suppressor cells (MDSCs) and Tregs [75, 76]. Exosomal miRNA miR-214 can target the tumor suppressor gene PTEN in T cells and induce immunosuppressive Tregs [77]. Tumor exosomes contribute to MDSC recruitment via chemoattractant chemokines CCL2 and CCL5 release [73, 78]. Moreover, tumor exosomes carry GM-CSF and IL-6, which promote MDSC expansion, survival, and an immunosuppressive state. The delivery of proteins, including arginase-1 and nitric oxide synthase (NOS2), through tumor exosomes further increases MDSCs' ability to suppress T cells [73].

Tumor exosomes often express the immune checkpoint protein, programmed death-ligand 1, on their surface. Upon binding to its receptor, PD-1, on T cells, PD-L1 inhibits T-cell proliferation, cytokine secretion, and cytotoxic activity. This interaction is a significant contributor to tumor immune evasion and the establishment of immune tolerance toward the tumor [79–82].

Tumor exosomes are crucial for pre-metastatic niche formation [83]. It has been shown that inhibiting exosome secretion can decrease melanoma lymph node metastasis in murine models [84]. Furthermore, elevated concentrations of tumor exosomes are often found in organs prone to metastasis; for instance, pancreatic cancer patients exhibiting high levels of tumor exosomes in the liver, a common site for pancreatic cancer metastasis, suggesting that exosomal content can be predictive of metastatic organotropism [85–87].

Tumor exosomes deliver matrix metalloproteinases (MMPs) and other proteases that remodel the extracellular matrix (ECM) at the pre-metastatic site, creating a conducive environment for tumor cell invasion [88, 89]. By transporting pro-angiogenic factors, vascular endothelial growth factor and basic fibroblast growth factor to endothelial cells and recruiting pro-angiogenic stromal cells, such as pericytes and fibroblasts, tumor exosomes support the development of a vascular network to sustain the growth of incoming metastatic cells [88, 90, 91]. Tumor exosomes also contribute to increased vascular permeability at future metastatic sites. The integrity of the vascular endothelial cell layer, normally maintained by adherens and tight junctions, can be compromised by tumor-secreted soluble factors and exosomes, leading to enhanced vascular permeability [87 , 92, 93]. This allows exosomal contents to access and transmit tumor-derived signals to local cells, thereby priming the tissue for the arrival of circulating tumor cells and facilitating entry of metastatic cells [94, 95].

The hypoxic conditions within the TME further support the pro-metastatic and immunosuppressive functions of tumor exosomes. Hypoxia stimulates the production and secretion of exosomes rich in immunoregulatory PD-L1, CSF-1, TGF-β, and hypoxia-inducible factor-1 alpha (HIF-1α). These intensify immune cell suppression and promote the recruitment and activation of immunosuppressive cells, including Tregs, MDSCs, and tumor-associated macrophages [96].

Organ-specific metastasis is also influenced by tumor exosomes through the expression of specific adhesion integrins on their surface. These integrins can bind to corresponding cell types or ECM components in recipient organs, thereby directing tumor cell recruitment and adhesion. Exosomal integrins α6β4 and α6β1 have been shown to promote lung adhesion, while αvβ5 directs liver tropism [87]. Other molecules, such as exosomal SMAD3, have been linked to increased reactive oxygen species production, enhancing tumor cell adhesion at metastatic sites [97]. CD146/MCAM on tumor exosomes, in conjunction with RalA/B GTPases, which control exosome biogenesis and organ targeting, has been implicated in lung metastasis [98].

### Roles of Treg exosomes in immunotolerance

Tregs, a subpopulation of CD4+ T cells, are responsible for maintaining immune homeostasis. Similar to tumor exosomes, Treg exosomes carry an array of biomolecules from proteins, miRNAs, and cytokines, that contribute to their immunomodulatory effects. Treg exosomes have been demonstrated to increase the secretion of anti-inflammatory cytokines IL-4 and IL-10, while decreasing pro-inflammatory cytokines IL-6, IL-2, and IFN-γ, leading to low inflammatory responses and immune tolerance [99].

In dendritic cells, Treg exosomes induce a tolerogenic phenotype, characterized by increased IL-10 and decreased IL-6 secretion [100]. Specific miRNAs within Treg exosomes confer their immunosuppressive abilities. miR-150-5p and miR-142-3p can be transferred from Treg exosomes to dendritic cells, where they induce a tolerogenic state and reduce the release of inflammatory cytokines [100]. In the context of inflammatory bowel disease, miR-195a-3p, found in Treg exosomes, targets the pro-apoptotic protein Caspase 12, thereby inhibiting apoptosis and alleviating disease severity [101]. Furthermore, miR-709 and miR-2861 carried by Treg exosomes play roles in neuroprotection and tissue repair in spinal cord injury, primarily by modulating local inflammatory responses and promoting recovery processes [47, 102]. In autoimmune multiple sclerosis, exosomal miRNA let-7i can target the TGFBR1 and IGF1R pathways, thereby suppressing the induction of pathogenic T-cell responses [103].

The protein content of Treg exosomes also contributes to their immunosuppressive action. iNOS, an enzyme involved in blocking cell cycle progression and inducing apoptosis in T cells, has been identified in Treg exosomes [104]. The ectonucleotidases CD39 and CD73, which are expressed by Treg cells, are also present in their exosomes and catalyze the hydrolysis of ATP to adenosine, an immunosuppressive molecule that promotes M2 macrophage polarization and facilitates tissue repair [105, 106]. Moreover, exosomes from dendritic cells engineered to express FOXP3, the master transcriptional regulator of Treg cells, have been shown to inhibit CD4+ T-cell proliferation, as well as reduce the production of pro-inflammatory cytokines IFN-γ, IL-6, and IL-17 [107]. Additionally, Treg exosomes can activate the PI3K/Akt signaling pathway in microglia, protecting against oxygen–glucose deprivation/reperfusion-induced injury [108].

Thus, Treg exosomes and tumor exosomes represent a mechanism wherein a complex interplay of cytokines, miRNAs, and proteins converges on specific signaling pathways to establish an immunosuppressive state.

## Exosomes in pathogen interactions and immune evasion

Exosomes play modulatory roles in immune inhibition of the body's first-line defense network through cellular crosstalk via EV signaling. They are important in the homeostatic control of immune activity, including immunosuppression, which relies on the source of the cell-derived molecular cargo [109]. Pathogenesis-linked cellular changes by viruses, bacteria, or parasites affect over 100 million people worldwide [110]. Exosomes can amplify the process of microbial pathogenesis, making network interplay between pathogens and host cells by directly transmitting PAMPs and indirectly influencing the infection kinetics by modulating the processes, such as immune evasion [109].

Certain infectious agents evade their host immune system with the support of exosomes, and this enhances their transmission. Infected cells release exosomes that modulate the body's immune defenses. This promotes the progression of infectious diseases [111] and enables cellular evasion from being detected by the body's immune defenses. This can occur at all phases of the infectious process [67, 112–114]. The mechanism adopted is when exosomes release complete virus particles and associated molecular constituents. This provides a distinct pathway that avoids damage to the cellular structure. This damage includes lysis and confers resistance to antibody-mediated neutralization [115–117]. Select pathogen-derived molecules and host cell factors package exosomes that might also participate in the disease process. This is revealed in pathogen miRNA and viral nucleic acids [118–120]. It is shown that tenascin-C, which is involved in fibrosis during COVID-19, plays a role in pathogenesis [121].

### Viral, bacterial, and parasitic manipulation of exosomal pathways

Studies show that the main target of viruses is to favor the expression of virulence-associated genes. During viral attacks, exosomes carry viral components, such as proteins, microRNAs, and mRNAs, which are transported to the target cells. Exosomes produced by human T-cell leukemia virus-1 (HTLV-1)-infected T-cell lines deliver the viral protein that enhances gene expression, which can activate transcription in target cells [116]. The exosomes released by cells infected with human immunodeficiency virus-1 (HIV-1) and HTLV-1 carry both viral and host cell proteins that prevent cell migration, along with dsRNA/ssRNA, which can boost the expression of nuclear genes and facilitate viral progression [122]. Evidence also suggests that exosomes associated with HIV-1 transactivator of transcription (TAT) could induce neurite shortening and neuron death [123].

In case of bacteria, exosomes released by *Staphylococcus aureus* contain a pore-forming molecule α-toxin, which functions as a key bacterial effector protein, therefore secreting it to distant cells [124]. Likewise, exosomes from Bacillus anthracis-infected cells function as carriers for key virulence-associated toxins to distant regions from the primary infection [125]. Furthermore, secretion of exosomes from cytotoxin-associated gene A (CagA)-exhibiting gastric epithelial cells travel through the bloodstream and distribute CagA, toxins, and pathogenic molecules to remote tissues and organs. Research indicates that the delivery of CagA is involved in the extra-gastric disorders associated with *Helicobacter pylori* [126].

Exosomes from parasites could play a role in pathogenicity and cell toxicity. For instance, research shows that nanotube-derived EVs from the bloodstream form of *Trypanosoma brucei* fuse with the host surface of RBCs, with fusion occurring through an unidentified EV surface protein. Fusion leads to the transfer of lipids and antigens unique to parasites, including the immunogenetic variant surface glycoprotein, to the red blood cell membrane. The structural characteristics of the RBC membrane are modified by this interaction. This results in the clearance of infected erythrocytes by macrophages in the spleen and liver [126]. It has also been revealed that *Toxoplasma gondii* can alter host cell (L6 cells) proliferation mechanisms by increasing the number of cells in the S phase. Exosomes strengthen this response by delivering molecular cargo to uninfected neighboring cells [126]. Notably, researchers discovered that exosomes derived from mature red blood cells during malaria infection carry a functional RNA-induced silencing complex with Argonaute 2, which could precisely inhibit gene expression in endothelial cells to modify the membrane integrity, thus supporting malarial infection [127].

Exosomes derived from intracellular parasites have some potential outcomes. *Leishmania* exosomes' effect on macrophages could suppress the body's first-line defenses [128–132]. *Trypanosoma cruzi* may mediate how the pathogen and host interact, altering immune function [133, 134]. During Plasmodium infection and transmission of disease-causing components, the transport of resistance-conferring proteins may enhance transmission [135, 136]. Exosomes modify immune responses, bidirectional pathogen–host interactions, and enhance binding capacity. This is found within the category of extracellular parasitic protozoa like *Trichomonas vaginalis* exosomes [137, 138].

### Exosomal packaging of pathogen molecules and immune diversion

Certain infectious agents evade their host immune system with the support of exosomes, and this enhances their transmission. For instance, the release of the hepatitis A virus from cells following attachment within host cell membranes protects the virion from antibody-triggered pathogen suppression. So, an exosomal pathway evades detection of the virus with antibody-mediated neutralization and gives a chance for a better virus to spread. The proteins vacuolar protein sorting 4 homolog B (VPS4B) and ALIX are key contributors to this mechanism [139]. Of particular interest was the observation that exosomes from virus-infected cells can disseminate miRNAs of non-host origin, making them evade detection by the host immune system [140] (Table 2).

Parasite–parasite signaling and parasite-host interaction allow them to bypass the host's defenses. The evidence shows *Trypanosoma brucei rhodesiense* exploits EVs to transmit serum resistance-protein (SRA) to *T. brucei*. The mechanism by which SRA works is to interact with complement proteins, preventing membrane attack complex formation. SRA is needed to evade the mechanism of host lytic factors, therefore, enabling the potential to evade innate immunity [141]. Furthermore, studies revealed that HIV-1-infected cells exploit exosomes to unlock HIV-1 production infection within quiescent primary CD4 + T lymphocytes [142]. Moreover, nematode parasites secrete exosomes by downregulating the key mediators (IL-33). This is achieved by inhibiting innate type 2 immunity *in vivo* [143]. It is also found that exosomes from *T. cruzi* activate a Th2 host immunity polarization through elevating effector molecule release of IL-4 and IL-10. Suppressing inducible pathways of NOS2 expression in CD4+ T cells and macrophages [133, 144].

Studies revealed that hepatitis B viral X protein (HBx) can promote the exosomal secretion of apolipoprotein B mRNA-editing catalytic polypeptide-like protein 3G or simply APOBEC3G. Since APOBEC3G inhibits HBV replication, a decrease in its intracellular level will favor infection [145]. Hepatitis B virus-infected hepatocytes produce exosomes that transport miR21 and miR-29a, which results in a downregulation of IL-12p35 mRNA expression, in turn leading to a constrained host innate immune response [146]. Table 1 summarizes the roles of exosomes in a range of viral, bacterial, and parasitic infections, while Table 2 expands on mechanisms of exosome-facilitated immune evasion.

**Table 1:** Pathogen-specific exosome manipulation and immune evasion.

Category	Pathogen	Exosome cargo/components	Target cells/process	Mechanism of action	Immune evasion strategy	References
**Viruses**	HTLV-1	Viral proteins, mRNAs	Host T-cells	Enhances gene expression, activates transcription	Facilitates viral progression	[116]
	HIV-1	Viral/host proteins, dsRNA/ssRNA, TAT protein	T-cells, neurons	Boosts nuclear gene expression; induces neural shortening and neuron death	Prevents cell migration, neurotoxicity	[122, 123]
	Hepatitis A	Whole virions	Host cells	Binds host membranes; uses VPS4B/ALIX proteins	Avoids antibody neutralization	[139]
	Hepatitis B	HBx protein, miR-21, miR-29a	Hepatocytes, immune cells	Downregulates APOBEC3G (antiviral protein); suppresses IL-12p35	Constraints on the innate immune response	[145, 146]
**Bacteria**	*Staphylococcus aureus*	α-toxin	Distant host cells	Pore-forming effector protein secretion	Cytotoxicity, systemic spread	[124]
	*Bacillus anthracis*	Toxin virulence factors	Remote tissues	Transport toxins from the infection site	Promotes systemic infection	[125]
	*Helicobacter pylori*	cagA toxin, pathogenic molecules	Gastric/epithelial cells, remote tissues	Bloodstream distribution of toxins	Cause extra-gastric disorders	[126]
**Parasites**	*T. brucei*	Variant surface glycoprotein, lipids	RBCs	Modifies RBC membrane structure	Clears infected RBCs via spleen/liver macrophages	[141]
	*T. gondii*	Proliferation signals	L6 cells, neighboring cells	Increases S-phase cells, delivers cargo to uninfected cells	Enhances host cell manipulation	[147]
	Malaria parasite	Argonaute 2-RISC complex	Endothelial cells	Inhibits gene expression; modifies membrane integrity	Supports infection persistence	[127]
	*Leishmania* spp.	Immunomodulatory molecules	Macrophages	Suppresses first-line defenses	Damage immunity	[128, 129, 130, 131, 132]
	*T. cruzi*	IL-4, IL-10	CD4 + cells, macrophages	Induces Th2 polarization; suppresses iNOS	Inhibits type 2 immunity	[133, 144]
	*T. vaginalis*	Adhesive factors	Host epithelial cells	Enhances binding capacity	Facilitates host–pathogen communication	[137, 138]

**Table 2:** Exosomal packaging strategies and immune diversion pathways.

Strategy	Pathogen example	Molecular components	Biological effects	Key proteins/pathways	Outcome	References
**Viral component packaging**	HIV-1, HTLV-1	Viral proteins, RNAs	Facilitates viral gene expression and cell-to-cell spread	TAT protein, dsRNA/ssRNA	Neuronal damage, immune evasion	[116, 122, 123]
**Antibody evasion**	Hepatitis A	Whole virions	Shield virus from neutralizing antibodies	VPS4B, ALIX	Enhanced viral transmission	[139]
**miRNA dissemination**	Virus-infected cells	Non-host miRNAs	Masks pathogen presence from immune detection	Viral miRNAs	Immune system evasion	[140]
**Complementary inhibition**	*T.b rhodesiense*	Serum resistance protein (SRA)	Blocks membrane attack complex formation	Complement proteins	Evade innate immunity	[141]
**Cytokine manipulation**	*T. cruzi*	IL-4, IL-10	Polarizes immunity to Th2 response, suppresses iNOS	CD4 + cells, macrophages	Inhibits antimicrobial defenses	[133, 144]
**Antiviral factor removal**	Hepatitis B	APOBEC3G	Reduces intracellular antiviral defenses	HBx protein	Promotes HBV replication	[145]
**Immune suppression**	Nematode parasites	IL-33 downregulation	Inhibits innate type 2 immunity	IL-33 pathway	Sustains infection	[143]
**Toxin delivery**	*S. aureus, B. anthracis*	α-toxin, virulence toxins	Induces cytotoxicity or systemic spread	Pore-forming proteins	Tissue damage, immune evasion	[124, 125]
**Host gene silencing**	*Malaria* parasite	Argonaute 2-RISC	Inhibits endothelial gene expression	RNA-induced silencing complex	Modifies host cell membranes	[127]
**Cross-species signaling**	*T. brucei*	Variant surface glycoprotein	Transfers parasite antigens to RBCs	EV surface proteins	Immune clearance avoidance	[141]

## Perspectives: from recent discoveries to future potentials in immunotherapy

### Targeting PD-L1 exosome loading abrogates CD8+ T-cell exclusion and offers a novel approach to overcoming immune desert

The study by Guan *et al*. [7] unveils a novel mechanism by which tumor cells evade immune surveillance, offering a promising avenue for improving cancer immunotherapy. The research demonstrates that phosphorylation of HRS, a key component of the ESCRT complex involved in exosome biogenesis, restricts the infiltration of CD8+ T cells into tumors. Specifically, ERK-mediated phosphorylation of HRS promotes the selective loading of PD-L1 into exosomes, leading to immunosuppression and resistance to anti-PD-1 therapy. This finding highlights the importance of exosomal PD-L1 as a critical mediator of immune evasion and suggests that targeting HRS phosphorylation could enhance the efficacy of immune checkpoint blockade.

Future immunotherapy strategies could focus on developing small-molecule inhibitors that specifically block HRS phosphorylation. Such inhibitors, potentially in combination with existing anti-PD-1/PD-L1 therapies, could improve T-cell infiltration into tumors, overcome resistance, and ultimately enhance patient outcomes. Moreover, HRS phosphorylation status in tumor biopsies could serve as a predictive biomarker to identify patients most likely to benefit from this combination therapy. Further investigation into the signaling pathways regulating HRS phosphorylation and the specific mechanisms of PD-L1 loading into exosomes will be crucial for the rational design of effective therapeutics.

### Exosome-mediated mitochondrial transfer impairing antitumor immunity offers a novel approach for blocking ICI resistance

Another landmark discovery led by Professor Yosuke Togashi [148] has uncovered a novel mechanism of immune evasion, providing new insights into the complex interplay between cancer cells and the immune system. Targeting mitochondrial transfer and addressing the resulting mitochondrial dysfunction in TILs holds promise for improving the efficacy of cancer immunotherapies and ultimately improving patient outcomes. This groundbreaking work illuminates exosome-mediated mitochondrial transfer as a key mechanism of immune evasion, presenting new avenues for clinically relevant therapeutic interventions. By demonstrating that tumor cells transfer mtDNA-mutated mitochondria to TILs via exosomes, impairing T-cell function and promoting ICI resistance, this study underscores the exosome as a critical target for next-generation cancer immunotherapies. Future research should focus on developing strategies to block this intercellular transfer via targeted interventions, such as TNT inhibitors or specific disruption of exosome release mechanisms, ultimately restoring robust T-cell infiltration and function within the TME.

Furthermore, the identification of mtDNA mutations in tumor tissue as a predictive biomarker for ICI response has immediate clinical implications. Validating this finding in larger patient cohorts and developing rapid, cost-effective assays to detect these mutations will enable more precise patient stratification for immunotherapy. Ultimately, combining exosome-targeted therapies with ICIs or pursuing personalized exosome-based immunotherapies may overcome resistance and unlock durable responses, transforming the landscape of cancer treatment.

### Engineering exosomes for targeted antigen delivery

Leveraging the inherent targeting capabilities of exosomes, future immunotherapies could employ engineered exosomes to deliver TAAs directly to APCs, such as dendritic cells. This approach would bypass systemic administration of antigens, potentially minimizing off-target effects and maximizing the activation of tumor-specific T-cell responses. Tailoring the exosomal surface with targeting ligands that specifically bind to receptors on APCs could further enhance the efficiency of antigen delivery and subsequent T-cell priming.

### Reprogramming the tumor microenvironment

Exosomes offer a unique opportunity to reshape the immunosuppressive TME. Future research could explore loading exosomes with immunomodulatory molecules, such as cytokines (e.g. IL-12 and IFN-γ), checkpoint inhibitors, or STING agonists, to promote T-cell infiltration, activation, and cytotoxic activity within the tumor. Engineering exosomes to deliver antiangiogenic factors or MMP inhibitors could also disrupt the tumor vasculature and extracellular matrix, respectively, further enhancing T-cell access and antitumor efficacy.

### Personalized exosome-based immunotherapies

The inherent ability of exosomes to reflect the cellular source from which they originate opens the door for personalized immunotherapies tailored to individual patient profiles. Future strategies could involve isolating and engineering exosomes from a patient's own immune cells or tumor cells to create a personalized vaccine that elicits a robust antitumor response. By presenting a comprehensive repertoire of TAAs and/or by delivering immunostimulatory signals, these patient-derived exosomes could overcome tumor heterogeneity and enhance the effectiveness of immunotherapy. The analysis of patient-derived exosome cargo with biomarkers for immune disorders may give insights into the potential for early disease prevention with specialized medicine.

In summary, exosomes are pivotal mediators of immune regulation, influencing both tumor and pathogen responses through the delivery of proteins, nucleic acids, and lipids. Their roles in antigen presentation, immune checkpoint modulation, and intercellular communication underscore their significance in anticancer immunity and host defense. Emerging therapeutic strategies, including the targeting of PD-L1 exosome loading, blocking exosome-mediated mitochondrial transfer, and engineering personalized exosome-based immunotherapies, illustrate the translational potential of this knowledge. Collectively, understanding and manipulating exosome biology provides a promising avenue for enhancing antitumor immunity, overcoming immune deserts, and designing next-generation immunotherapeutic interventions.

## Data Availability

All data cited in this review are publicly available from the referenced publications.

## Abbreviations

ADE: ascites-derived exosome
ALIX: ALG-2-interacting protein X
APC: antigen-presenting cell
BMDC: bone marrow-derived dendritic cell
FDC: follicular dendritic cell
DAMP: damage-associated molecular patterns
DC: dendritic cell
DEX: dendritic cell exosomes
ECM: extracellular matrix
EDC: exosome-deficient dendritic cell
EMT: epithelial–mesenchymal transition
ESCRT: endosomal sorting complexes required for transport
EV: extracellular vesicle
GM-CSF: granulocyte-macrophage colony-stimulating factor
HRS: hepatocyte growth factor-regulated tyrosine kinase substrate
HSP: heat shock protein
iNOS: inducible nitric oxide synthase
ILV: intraluminal vesicle
IS: immunological synapse
LAM: lipoarabinomannan
LPS: lipopolysaccharide
MHC: major histocompatibility complex
miRNA: microRNA
MMP: matrix metalloproteinase
mRNA: messenger RNA
MSC: mesenchymal stem cells
MVB: multivesicular body
PD-L1: programmed death-ligand 1
PRR: pattern recognition receptor
RAGE: receptor for advanced glycation end products
SRA: serum resistance protein
TCR: T-cell receptor
TEMs: tetraspanin-enriched microdomains
TIL: tumor infiltrating lymphocytes
TLR: toll-like receptor
TNF: tumor necrosis factor
Treg: regulatory T cell
